# Supplementary material for: How doctors build community and socialize into a clinical department through morning reports. A positioning theory study
Source: PLoS One. 2023 May 9;18(5):e0284999. doi: 10.1371/journal.pone.0284999 (PMC10168562; doi:10.1371/journal.pone.0284999)
Supplement: Attachment — Submitted filename: Response to reviewers.docx [file pone.0284999.s001.docx]

Dear Editor

Thank you for your positive feedback on our revised version and the opportunity to revise the manuscript. We have tried to address all the comment and find that this strengthens the article.

Best regards

Jane Ege Møller

| Comment Reviewer/Editor | Answer |
| --- | --- |
| Ed: The absence of a raw data file is understandable when livelihoods and professional reputations are at stake. Consider adding a few more example excerpts if they make sense, and encourage readers to contact you with specific questions on the corpus. | We agree that a raw data file would have been ideal, and we thank for your understanding of why it is not possible We find it difficult to add example excerpts at this stage, however we agree to encourage readers to contact us. We have tried to add these in information we add in the editorial system. |
| Ed: In your final revision, please indicate all of the limitations and wording concerns that Reviewer 1 identifies, such as the absence of convergent evidence. | We have revised accordingly. Please se details below. |
| Ed: I agree that you can manage the generalization issue better, though I have a slightly different take on how to address this.   In my opinion, we could use a better description of the idiosyncracies of the environments that you observed.  I feel like we are missing context, which could be provided in your setting description.  I don't understand the general admission procedure and hence the doctor's defensive response to being challenged.  I surely don't understand the availability of hospital beds in Denmark, and how close to the edge of capacity the Danish system functions.  I have no insight into the prevalence of administrative intrusion that motivated the humorous exchange. | Thank you for this suggestion. We have tried to clarify the context by adding the following:  To better understand our study, it is relevant to know that the healthcare system in Denmark is primarily public, and general practitioners are gatekeepers for secondary and tertiary care. As a citizen, you need a referral from your general practitioner to be admitted to a hospital. The hospitals have limited capacity, and there are generally waiting lists. However, by law, various “packages of care” have been introduced. Thus, a patient with symptoms of serious or acute illness (for example cancer) can bypass the waiting list in order to be treated fast. Likewise, there are rules ensuring that as a patient, your symptoms will be considered within 30 days from referral. The hospitals are financed by one of five regions and therefore, the regional authorities prefer to keep treatment inside the region, since treatment in another region would entail extra payment. There can be an inbuilt conflict between doctors, who want the best treatment for their patients, and the management, who wants the most treatment for the finances they have at disposal from the government.. |
| Rev 1: In my view, the limitations of the study are not pointed out well enough. A major limitation is that recordings without interviews do give any information about intentions nor about effects. In your previous version, it was clear that the temptation is great to make that kind of conclusions. And even in this version, there are some left. You say that, according to Kvale, interpretations can be theory based. However, in social science such theoretical interpretations do not establish facts, and what is said should remain recognizable as interpretation (f. i. by stating “this can be seen as; or “this can be expected to lead to…”, and not using an indicative (they do, the feel…) or “this establishes” or “this leads to”. This is something that still needs correction in a few places. | We have carefully read through the manuscript and changed the (two) places were this could be misinterpreted. If we are acquired to revise this point further we would need specific guidance as to where in the text. |
| Rev 1: Generalization is an issue. You say that your findings are not generalizable since you examined only two hospitals. To begin with you pointed out that the morning reports in Denmark have a specific form. Therefore, transferability to other countries is low (assuming that in all Danish hospitals the format, intentions and practice are similar). Generalizability cannot be obtained as in no way saturation is achieved (and theory development minimal). In my view, it would be more correct to say that it is uncertain whether the findings portray what is the case in other settings as it is unclear whether the existing variation has been captured. | We have tried to addressed these points in the discussion in the section that now reads:  Our study has limitations. Studies have shown that registrars often criticize the tone, leadership, and learning environment of morning reports [1]. Our study does not elucidate this dimension, as we have only observed the performed actions, and not asked participating doctors about their perspective. In addition, interviews would have shed light on the intentions behind and perceived effects of the interactions which was beyond the scope of this observation-based study. Methodologically, the use of video observations raises the question about participant reactivity, i.e. if the method caused changes in participants’ behaviors [45]. However, in our continued dialogue with participants during the data collection, we did not hear any reports about the morning report being different than usual. A limitation is that our study took place in a limited number of departments in Denmark at only two different hospitals. This prevented us from achieving full data saturation. We found repeated interactions and variations, but we cannot rule out the possibility that new themes would have appeared had we conducted more observations. Furthermore, Denmark has a “flatter” hierarchy than other countries and thus the results might not be generalizable. Still, our comprehensive data material ensured rich data, and the use of video observation where all authors participated in the analysis enabled us to revisit the recordings several times in order to validate the analysis. In addition, we provide a theoretical development by using the framework of positioning theory to gain new insights into the collegial interactions among hospital doctors. This was strengthened by the interdisciplinary group of researchers from the humanities and medicine, allowing for multiple perspectives that enriched the analysis, which is a recommended research strategy [7] |
